# Characterization of an IncR Plasmid with Two Copies of IS*CR*-Linked *qnrB6* from ST*968 Klebsiella pneumoniae*

**DOI:** 10.1155/2020/3484328

**Published:** 2020-11-24

**Authors:** Changrui Qian, Xinyi Zhu, Junwan Lu, Kai Shen, Qianqian Chen, Wangxiao Zhou, Hongmao Liu, Wei Lu, Danying Zhou, Zhewei Sun, Xi Lin, Kewei Li, Qiyu Bao, Teng Xu, Shunfei Lu

**Affiliations:** ^1^Key Laboratory of Medical Genetics of Zhejiang Province, Key Laboratory of Laboratory Medicine, Ministry of Education, China, School of Laboratory Medicine and Life Sciences, Wenzhou Medical University, Wenzhou 325035, China; ^2^Institute of Biomedical Informatics, Wenzhou Medical University, Wenzhou 325035, China; ^3^Center of Prenatal Diagnosis, Jiaxing Maternity and Child Health Care Hospital, Jiaxing 314000, China; ^4^Institute of Translational Medicine, Baotou Central Hospital, Baotou 014040, China; ^5^School of Medicine and Health, Lishui University, Lishui 323000, China

## Abstract

To characterize the molecular structure of IncR plasmid-related sequences, comparative genomic analysis was conducted using 261 IncR plasmid backbone-related sequences. Among the sequences, 257 were IncR plasmids including the multidrug-resistance IncR plasmid pR50-74 from *Klebsiella pneumoniae* strain R50 of this work, and the other four were from bacterial chromosomes. The IncR plasmids were derived from different bacterial genera or species, mainly *Klebsiella pneumoniae* (70.82%, 182/257), *Escherichia coli* (11.28%, 29/257), *Enterobacter cloacae* (7.00%, 18/257), and *Citrobacter freundii* (3.50%, 9/257). The bacterial chromosomes carrying IncR plasmid backbone sequences were derived from *Proteus mirabilis* AOUC-001 and *Klebsiella pneumoniae* KPN1344, among others. The IncR backbone sequence of *P. mirabilis* AOUC-001 chromosome shows the highest identity with that of pR50-74. Complex class 1 integrons carrying various copies of IS*CR1-sdr-qnrB6-△qacE/sul1* (IS*CR1*-linked *qnrB6* unit) were identified in IncR plasmids. In addition to two consecutive copies of *qnrB6*-*qacE*-*sul1*, the other resistance genes encoded on pR50-74 are all related to mobile genetic elements, such as IS*1006*, IS*26*, and the class 1 integron. This study provides a clear understanding of the mobility and plasticity of the IncR plasmid backbone sequence and emphasizes the important role of IS*CR* in the recruitment of multicopy resistance genes.

## 1. Introduction

IncR was first characterized as a new plasmid incompatibility group in 2009 [1]. The first sequenced IncR single-replicon plasmid was pEFER, though IncR often coexists with other replicons such as IncC, IncN, IncHI, and IncFII [2]. There are an increasing number of reports about IncR plasmids carrying various resistance genes, such as *bla_KPC-2_*, *bla_DHA-1_*, *bla_NDM-1_*, *bla_VIM-1_, qnrS1*, or *armA*, in clinical Enterobacterales, especially in *Klebsiella pneumoniae* strains from different geographical regions [3–5]. Importantly, the pool of resistance genes carried by IncR replicon plasmids may spread to transferable plasmids through transposition or plasmid recombination events, contributing to the high plasticity of multiple-replicon plasmids [2].

Integrons and insertion sequence common region (IS*CR*) elements were originally named common regions (CRs), which represent a family of mobile genetic elements (MGEs) that are similar to the insertion sequence (IS) element [6]. IS*CR* can transpose adjacent DNA sequences by rolling circle replication, and this process has been demonstrated experimentally using MGEs IS*91* and IS*1294* [7]. IS*CR* elements are notable for their close association with a wide variety of antibiotic resistance genes. Complex class 1 integrons are large, flexible genetic elements that consist of different class 1 integrons followed by IS*CR* [8]. As complex class 1 integrons are powerful gene-capturing tools that can mobilize extremely large sections of DNA encoding a variety of resistance genes related to chloramphenicol, trimethoprim, quinolone, and *β*-lactam antibiotics [7], these elements play important roles in the recruitment and spread of resistance genes.

Based on sequencing of a multidrug-resistance IncR plasmid from a *K. pneumoniae* strain carrying a complex class 1 integron with two consecutive IS*CR1*-linked *qnrB6* units, comparative genome analysis of the IncR plasmid with various structures of the IS*CR1*-related complex class 1 integron was performed in this study.

## 2. Materials and Methods

### 2.1. Bacterium Genome Sequencing and Annotation

*K. pneumoniae* R50 was isolated from an anal swab sample of a rabbit from a farm in Wenzhou, South China. Genomic DNA was extracted from *K. pneumoniae* R50 using an AxyPrep Bacterial Genomic DNA Miniprep Kit (Axygen Scientific, Union City, CA, USA) and sequenced by Illumina HiSeq-2500 and Pacific Bioscience sequencers at Annoroad Gene Technology Co., Ltd. (Beijing, China). Sequencing reads of approximately 10-20 kb in length were assembled with Canu v1.7 [9]. Error correction of tentative complete circular sequences of the plasmids and the chromosome was performed using Pilon version 1.18 [10] with two FASTQ read sets derived from the HiSeq-2500 sequencing platform.

Open reading frames (ORFs) and pseudogenes were predicted using Prokka with default parameters, combined with BLASTP searches against the UniProtKB/Swiss-Prot and RefSeq databases [11–14]. The rRNA gene sequences were annotated by RNAmmer [15], and the tRNA sequences were annotated by tRNAscan-SE 2.0 [16]. Annotation of resistance genes, mobile genetic elements, and other features was carried out using online databases including CARD [17], ISfinder [18], INTEGRALL [19], ResFinder [20], and the Tn Number Registry [21]. The number of iteron tandem repeats was predicted by Tandem Repeats Finder [22]. Gene organization diagrams were drawn in Inkscape 0.48.1 (https://inkscape.org/en/).

### 2.2. Antimicrobial Susceptibility Testing

Minimum inhibitory concentration (MIC) assessment of 16 antimicrobial agents was performed using the agar dilution method in accordance with the guidelines of Clinical and Laboratory Standards Institute (CLSI document M100-S27, 2017).

### 2.3. Comparative Genomics and Phylogenetic Analysis

Plasmid pHN84KPC (KY296104) was selected as reference because it contains the most complete IncR backbone, and gene sequences with coverage > 50% and identity > 80% to those of pHN84KPC were considered effective homologous backbone genes [5]. Sequences containing the backbone genes *resD*, *repB*, *parAB*, and *umuCD* were used for the phylogenetic analysis. Multiple sequence alignments were performed using MAFFT v7 [23], and an unrooted maximum likelihood phylogenetic tree was reconstructed using the generalized time-reversible model of evolution with uniform rates of substitution in MEGA7 [24]. The phylogenetic tree was visualized using the interactive web platform iTOL [25]. The sequence retrieval, statistical analysis, and other bioinformatics tools used in this study were applied with python scripts.

### 2.4. Nucleotide Accession Numbers

The complete sequences of the *K. pneumoniae* R50 chromosome (CP040362) and plasmid pR50-74 (CP040363) have been submitted to NCBI GenBank.

## 3. Results and Discussion

### 3.1. Main Features of *K. pneumoniae* R50

The *K. pneumoniae* R50 genome consists of one 5,210,287 bp circular chromosome encoding 4236 ORFs and one plasmid (named pR50-74) of 74,011 bp in length encoding 89 ORFs (Table S1). Based on analyses of seven housekeeping genes, including *gapA*, *infB*, *mdh*, *pgi*, *phoE*, *rpoB*, and *tonB* [26], *K. pneumoniae* R50 was classified into the multilocus sequence type ST948. To the best of our knowledge, this is the first sequenced *K. pneumoniae* strain of this multilocus sequence type. R50 was further classified into the K5 capsular serotype based on the capsular locus of the *wzi-5* allele. A total of 13 antibiotic resistance genes are encoded in the *K. pneumoniae* R50 genome, with 4 and 9 in the chromosome and the plasmid pR50-74, respectively. The resistance genes are mainly related to the antibiotics of florfenicol, tetracycline, quinolone, aminoglycoside, rifampicin, trimethoprim, streptomycin, and sulfonamides (Table 1), in accordance with the results of antimicrobial susceptibility testing of the bacterium (Table 2).

### 3.2. Structural Characterization of IncR Plasmid Backbone Sequences

The backbone sequences of the most sequenced IncR plasmids usually contain *vagCD* (toxin-antitoxin system), *resD* (multimer resolution), *repB* (replication initiation), *parAB* (partition), *umuCD* (stability), and *retA* (maintenance) genes [2]. Plasmid pR50-74 with two replicons, R and FIA(HI1), carries seven of these genes but lacks *vagCD*, which are generally located upstream of *resD* on IncR plasmids.

**Figure 1:**
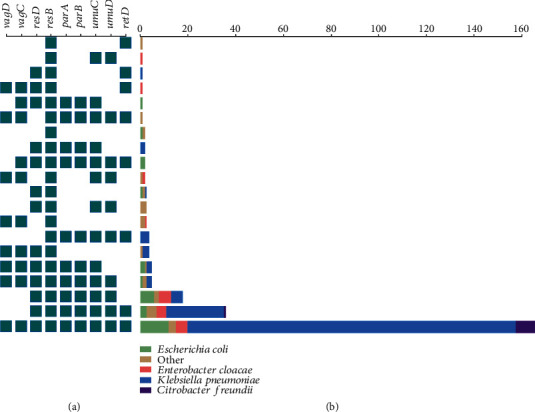
Distribution of plasmids with different numbers of backbone genes. The small squares on the left represent the presence of corresponding backbone genes. (b) The bars represent the number of plasmids and the corresponding species.

To characterize the presence of backbone genes among IncR plasmids, we used the single-replicon plasmid pHN84KPC as a reference because it contains the most complete IncR backbone regions [27]. A total of 261 genome sequences containing the IncR replicon gene *repB*, showing >90% identity and >80% coverage with that of pHN84KPC, were retrieved from GenBank. The range of identity and coverage of *repB* gene in 261 sequences to that of pHN84KPC was 93.6%-100% and 98%-100%, respectively. Among these 266 sequences, 257 are from plasmids and 4 from chromosomes. The plasmid sequences are from 20 different species: *K. pneumoniae* (70.82%; 182/257), *Escherichia coli* (11.28%; 29/257), *Enterobacter cloacae* (7.00%; 18/257), *Citrobacter freundii* (3.50%; 9/257), and 16 other species (8.17%; 21/257) (Table S2). In addition to *repB*, other backbone genes were also screened. Approximately 64.20% (165/257) of the plasmids contain all 9 backbone genes; 14.00% (36/257) of the plasmids only lack *vagCD* genes, and 7.00% (18/257) of the plasmids lack both *vagCD* and *retA* genes (Figure 1). The results demonstrate that *vagCD* and *retA* are less conservative than other backbone genes. Interestingly, among the 4 chromosome sequences, one from *K. pneumoniae* KPN1344 (CP033901) and one from *Proteus mirabilis* AOUC-001 (CP015347) harbor all 9 backbone genes; the two from *E. cloacae* strains 339389L (CP026536) and FDAARGOS_77 (CP026975) only contain 3 or 5 backbone genes, respectively.

**Figure 2:**
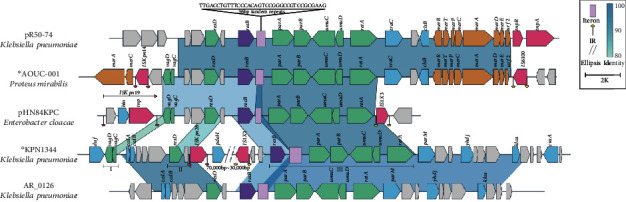
Linear comparison of the backbone regions of pR50-74, AOUC-001, PHN84KPC, KPN1344, and AR_0126. Genes are denoted by arrows. Genes, mobile elements, and other features are colored based on functional classification. The colors of the shadow areas represent different identities (80%-100%). Numbers under double diagonal indicate nucleotide positions for corresponding plasmids. Chromosome sequences are marked with asterisks.

The IncR backbone region of AOUC-001 and pR50-74 were almost identical (95% BLAST coverage and 99% nucleotide identity) to each other. However, the IncR backbone sequence in the KPN1344 chromosome can be separated into three parts, *vagCD*, *resD*, and *repB-parAB-umuCD-retA*, among which the second and third parts are approximately 40 kb apart (Figure 2). Comparison result of the sequence of these three parts combined with the 257 IncR plasmids mentioned above revealed that the highest homologous sequence to it was the backbone sequence of plasmid AR_0126 (CP021741) from *K. pneumoniae* (Figure 2). Further comparative genomics analysis was applied to AOUC-001, pR50-74, pHN84KPC, KPN1344, and AR_0126. The backbone region of pHN84KPC is flanked by two IS elements, IS*Kpn19* and IS*1X3*, which may promote the mobility of the sequence. AOUC-001 and pR50-74 share a nearly identical backbone sequence, except that the latter lacks *vagCD* genes. Furthermore, an identical *traC-clsB*-mercury operon sequence downstream of the *retA* gene of both AOUC-001 and pR50-74 is also present on other IncR plasmids, such as pKP6402 (AP018752) from *K. pneumoniae* (Figure 2). The backbone sequence of AOUC-001 is flanked by IS*Kpn14* and IS*6100*; however, no adjacent direct repeat sequence was identified. Nonetheless, *vagCD* genes are missing from the plasmid AR_0126, and the region upstream of *resD* is an approximately 3 kb fragment harboring *ccdAB*, which is different from pHN84KPC and other IncR plasmids. This result suggests that the IncR plasmid backbone sequence in KPN1344 and AR_0126 may have derived from the same ancestor and underwent deletion of *vagCD* as well as insertion of a 40 kb fragment between *resD* and *repB* during evolution.

**Figure 3:**
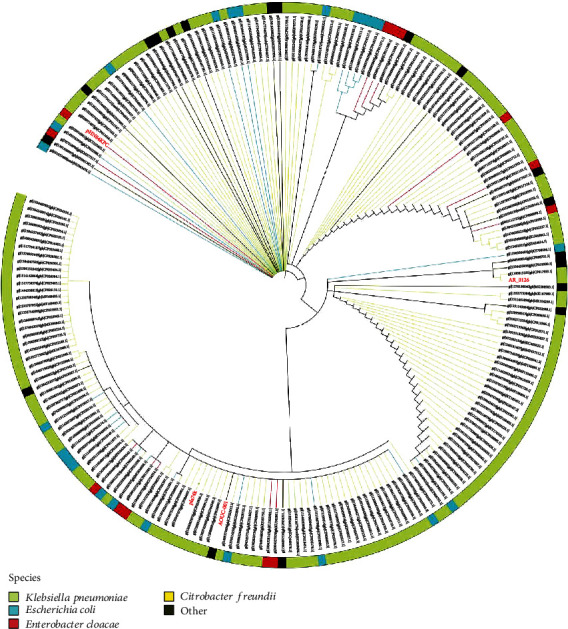
Phylogeny of backbone sequences of IncR plasmids.

The sequence differences in IncR backbones among pHN84KPC, pR50-74, KPN1344, AOUC-001, and AR_0126 are mainly in the regions encoding *vagCD*, *resD*, *repB*, and iterons which are direct repeat DNA sequences that play an important role in regulating plasmid copy number in bacterial cells [28]. The iteron regions of these five sequences are all composed of the same 36 bp tandem repeats, only with repeat number difference (Figure 2). The numbers of tandem repeats for pHN84KPC, pR50-74, KPN1344, AOUC-001, and AR_0126 were 10.5, 15.5, 22.5, 15.5, and 16.5, respectively. The identities of the *vagCD* genes on KPN1344 and AOUC-001 compared to those on pHN84KPC are 86% and 89%, respectively. In addition, the identities of the *resD* genes of pR50-74, KPN1344, AOUC-001, and AR_0126 compared to those of pHN84KPC are 100%, 86%, 100%, and 88%, respectively. Moreover, phylogenetic analysis of concatenated backbone genes *resD*, *repB*, *parAB*, and *umuCD* among 215 sequences (Table S3), including the two chromosomes, indicated that the IncR backbone sequence of AOUC-001 is evolutionarily closer to that of pla74k (Figure 3). It could be deduced that the IncR backbone of pR50-74 and AOUC-001 likely has the same origin. It was previously reported that MGEs, like Tn*133*1, mediate the evolution of type B plasmids carrying IncN3-like segment from type A plasmids carrying IncR backbone [29]. Papagiannitsis et al. reported that an MDR plasmid pKP1780 carries IncR backbone segment flanked by two-copy IS elements [30]. The MGEs on both sides of the backbone enable extensive reorganization among plasmids and chromosomes.

### 3.3. Comparative Analysis of the MDR Region of pR50-74-Related IncR Plasmids

**Figure 4:**
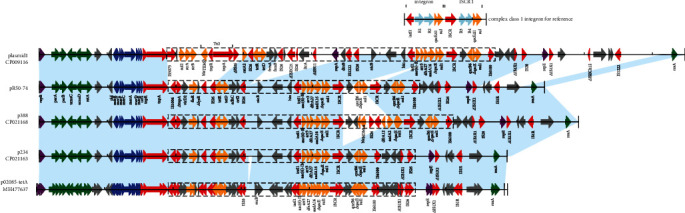
Comparative analysis of the genome structures of pR50-74, plasmid1, p388, p234, and p02085-tetA. Genes are denoted by arrows. Genes, mobile elements, and other features are colored based on functional classification. Shading denotes homologous regions (>95% nucleotide identity).

The multidrug resistance (MDR) region of pR50-74 carries *floR*, *tetD*, *qnrB*, *aac(6*′*)-Ib-cr5*, *arr3*, *dfrA27*, *aadA16*, and *sul1* (Figure 4), conferring resistance to chloramphenicol/florfenicol, tetracyclines, quinolones, aminoglycosides, rifampicin, trimethoprim, streptomycin, and sulfonamides, respectively. The MDR region can be divided into three fragments, and all of the resistance genes are associated with MGEs (Figure 4). The first fragment is a transposon structure carrying a *lysR-floR-virD2* unit that is conserved among the majority of *floR*-related sequences and often shares the same upstream direct repeats (DRs) and a complete or truncated downstream *tnp* unit [31]. The second fragment is a complex transposon sequence bracketed by two IS*26* in opposite orientation containing *tetR*, *tetD*, *adhC*, and *orf2*, which are commonly found on other plasmids such as those of two *Citrobacter* sp. strains, TA3 (AB257967) and TA6 (AB089600), and the *Alteromonas* sp. strain TA55 (AB089601) isolated from fish [32]. The last fragment is a complex class 1 integron carrying 4 resistance gene cassettes (*aac(6*′*)-Ib-cr5*, *arr3*, *dfrA27*, and *aadA16*) and two copies of resistance gene-related IS*CR1* units (IS*CR1-sdr-qnrB6-△qacE-sul1*).

Four plasmids sharing the highest nucleotide sequence similarities (coverage > 80%, identity ≥ 99%) with pR50-74 were retrieved from the NCBI nucleotide database. Two of the plasmids, p234 (CP021163, 68,573 bp) and p388 (CP021168, 79,064 bp) from *E. cloacae*, share the highest similarity (100% coverage and 99% identity) with pR50-74. Additionally, plasmid p02085-tetA (MH477637.1, 67,510 bp) from *C. freundii* shares 98% coverage and 99% identity with pR50-74; the last plasmid (CP009116, 94,760 bp) from the same species (*Klebsiella pneumoniae*) as pR50-74 shares low similarity (84% coverage and 99% identity) with pR50-74. The entire plasmid sequence of p02085-tetA is almost identical to that of p234, except for the absence of an approximately 1000 bp sequence between IS*26* and *nicB* (Figure 4).

The main difference between pR50-74 and p234 or p388 is a distinct structure of the complex class 1 integron (Figure 4). pR50-74 exhibits a tandem repeat sequence of two copies of the IS*CR1*-linked qnrB6 unit (ISCR1-*sdr-qnrB6-△qacE-sul1*) encoded carried by the integron, but p234 has only one copy of the unit. Interestingly, p388 has two different IS*CR1*-linked resistance gene arrays: one is the IS*CR1*-linked *qnrB6* unit, which is identical to the two units in pR50-74 units, and the other is *bla*_NDM-6_-IS26*-dfrA12-aadA2-△qacE-sul1*. As mentioned above, except for pR50-74, which carries two copies of the IS*CR1*-linked *qnrB6* unit, the other four plasmids have only one. Unlike pR50-74, the upstream region of the *tetR-tetD-adhC-orf2* fragment unit in plasmid1 (CP009116) shows insertion of the *sul2-strA-strB* unit fragment and a Tn*2* transposon carrying *bla_TEM_* (Figure 4).

### 3.4. Construction of the Multicopy IS*CR1*-Mediated Complex Class 1 Integron

The complex class 1 integron of pR50-74 can be divided into two MGE-associated units. One is a typical class 1 integron containing 5′CS (*intI1*-*attI*), 3′-CS (*qacE△1*-*sul1*-*orf5*), and a variable region of the gene cassettes (*aac6-arr3-dfrA27-aadA16*). The other is composed of IS*CR1-*linked *qnrB6* units. Using these two units as a query, 14 sequences containing either or both units of the complex class 1 integron were retrieved from GenBank. These sequences were further clustered into 4 groups. Group 1 includes six plasmids (CP030917, CP028582, CP028553, CP028717, CP019006, and CP018945) that only carry unit one. Group 2 includes six plasmids (MG874044, CP023950, CP009116, CP021163, MH477637, and CP031259) harboring both units. pR50-74, which carries two copies of IS*CR1*-linked qnrB6 unit, consists of group 3 alone. Group 4 is composed of two plasmids (MG870194 and CP030940), which carry four copies of IS*CR1*-linked qnrB6 unit, of which MG870194 (from *Salmonella enterica*) has a class 1 integron identical to that of pR50-74, though *aadA16* is absent from CP030940 compared to pR50-74 (Figure S1). No sequence containing three copies of the IS*CR1*-linked *qnrB6* unit was identified. These results suggest that the structure of the complex class 1 integron varies across bacterial species and that these complex class 1 integron sequences, including those with duplicated IS*CR1*-linked *qnrB6* units, share the same ancestor.

**Figure 5:**
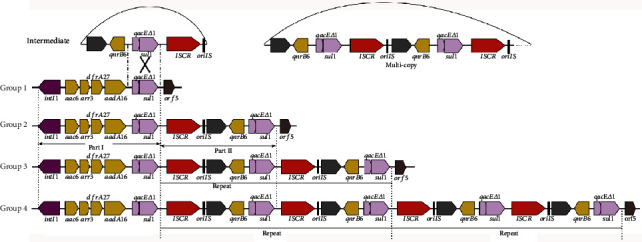
Four groups of sequence diagrams related to the formation of multiple copies of IS*CR1*-mediated complex class 1 integrons. The proposed circular intermediate of an aberrant RC replication carrying *sdr-qnrB6-△qacE-sul1* is shown above. The cross symbol represents a possible homologous recombination event. The sequence in the rectangle represents the starting point of the homologous sequences.

As a member of an extended family of IS*91*-like elements, IS*CR* elements lack terminal inverted repeats but have distinct terminal sequences designated as *oriIS* and *terIS* that indicate unique sites for the initiation and termination, respectively, of the rolling-circle (RC) replication stage of RC transposition [33]. However, there is a degree of inaccuracy with regard to recognition of *terIS*, causing replication to proceed beyond *terIS* and into the adjacent sequence. This feature confers IS*CR* with a critical role in bacterial genetics, and it may be responsible for the mobilization of class 1 integrons [34]. According to the model proposed by Toleman et al. [34], the construction of multicopy IS*CR1*-linked integrons can be explained in two steps. First, a circular intermediate that carries *sdr-qnrB6-△qacE-sul1* is produced by twice aberrant RC replication initiated from the replication origin *oriIS* of the IS*CR1* element. The circular intermediate is then inserted into the integron-like group 1 sequence by homologous recombination at the locus between 41 bp upstream of *△qacE* and 25 bp downstream of *sul1* (Figure 5). This process might occur once or more and generate a single- or multicopy IS*CR1*-linked complex class 1 integron. A similar process has been reported by Chen et al. [35], in which an IS*CR1*-linked *qnrB2* unit was inserted into an integron-IS*CR1* variant to produce an IS*CR1*-*qnrB2*-IS*CR1* structure.

## 4. Conclusion

The IncR plasmid backbone sequences were identified encoded on both the plasmids and chromosomes in bacteria of a variety of genera. It indicates the wide distribution of IncR plasmids and its backbone sequences. The resistance genes carried on IncR plasmids, such as pR50-74 of this work, are generally related with MGEs. Analyzing the molecular characterization of the complex class 1 integrons, which contain various copies of IS*CR1*-linked *qnrB6* units, allows for a clear understanding of the recruitment and dissemination of resistance genes mediated by MGEs, especially those by IS*CRs*.

## Figures and Tables

**Table 1:** Antibiotic resistance gene of R50.

Resistance to	Gene	Genetic context	Identity%
Beta-lactams	*bla_SHV-1_*	Chromosome	100
Aminoglycosides	*aadA16*	Plasmid	98
	*aac(6)-Ib*	Plasmid	95
Quinolones	*oqxA*	Chromosome	99
	*oqxB*	Chromosome	99
	*qnrB6*	Plasmid	100
Sulfonamides	*sul1*	Plasmid	100
Rifapentine	*arr3*	Plasmid	100
Trimethoprim	*dfrA27*	Plasmid	100
Tetracyclines	*tetD*	Plasmid	99
	*tetR*	Plasmid	99
Fosfomycin	*fosA*	Chromosome	96
Florfenicol	*floR*	Plasmid	99

**Table 2:** Antibiotic resistance profile of R50.

Drug	MIC (*μ*g/ml)	Phenotype
Ampicillin	32	Resistant
Spectinomycin	32	
Florfenicol	1024	Resistant
Chloramphenicol	512	Resistant
Nalidixic acid	32	Resistant
Gentamicin	≤0.25	Sensitive
Tobramycin	4	Sensitive
Tetracyclines	512	Resistant
Kanamycin	4	Sensitive
Streptomycin	4	Sensitive
Amikacin	≤1	Sensitive
Cephalothin	≤0.03	
Azithromycin	≤1	Sensitive
Sisomicin	2	
Rifampin	>1024	Resistant
Erythromycin	64	

## Data Availability

The data used to support the findings of this study are included within the article.

## Supplementary Materials

Supplementary Materials Supplementary Figure S1: complex class 1 integrons of group 3 (pR50-74) and group 4 (CP030940, MG870194). Table S1: general features of K. pneumoniae R50 genome. Table S2: species distribution of IncR plasmids. Table S3: bacterial information for evolutionary analysis.
