# Supplementary material for: Characterization of an IncR Plasmid with Two Copies of ISCR-Linked qnrB6 from ST968 Klebsiella pneumoniae
Source: Int J Genomics. 2020 Nov 24;2020:3484328. doi: 10.1155/2020/3484328 (PMC7707992; doi:10.1155/2020/3484328)
Supplement: Supplementary Materials — Supplementary Figure S1: complex class 1 integrons of group 3 (pR50-74) and group 4 (CP030940, MG870194). Table S1: general features of K. pneumoniae R50 genome. Table S2: species distribution of IncR plasmids. Table S3: bacterial information for evolutionary analysis. [file 3484328.f1.docx]

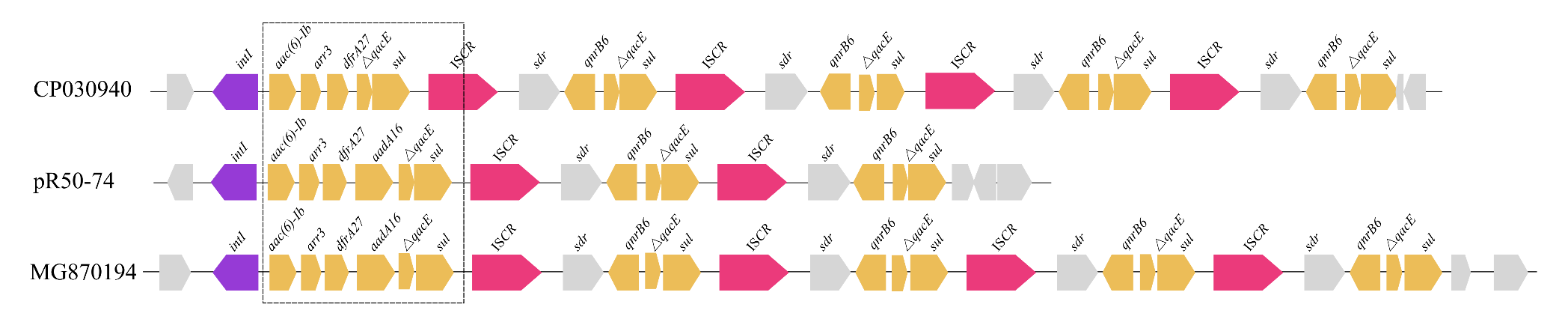


**Figure S1 complex class 1 integrons of Group 3 (pR50-74) and Group 4 (CP030940, MG870194)**

|  | chromosome | pR50-74 |
| --- | --- | --- |
| Size (bp) | 5210287 | 74011 |
| G+C % | 57.50 | 53.10 |
| Total opening reading frames | 4,792 | 89 |
| Known proteins | 4236 (88%) | 66 (74%) |
| Hypothetical proteins | 556 (12%) | 23 (26%) |
| Protein coding sequence (%) | 87 | 80 |
| Average ORF length (bp) | 947 | 665 |
| rRNA operons | 1*(16s-23s-5s-5s)  7*(16s-23s-5s) |  |
| tRNA | 91 |  |

**Table S1. General features of *K. pneumoniae* R50 genome**

**Table S2. Species distribution of IncR plasmids**

| **species** | **count** |
| --- | --- |
| Citrobacter farmeri | 1 |
| Citrobacter freundii | 9 |
| Citrobacter rodentium | 1 |
| Enterobacter asburiae | 2 |
| Enterobacter cloacae | 18 |
| Enterobacter hormaechei | 3 |
| Enterobacter roggenkampii | 1 |
| Escherichia coli | 29 |
| Escherichia fergusonii | 1 |
| Klebsiella aerogenes | 2 |
| Klebsiella oxytoca | 1 |
| Klebsiella pneumoniae | 182 |
| Klebsiella quasipneumoniae | 3 |
| Klebsiella variicola | 3 |
| Pantoea agglomerans | 1 |
| Proteus mirabilis | 1 |
| Providencia stuartii | 1 |
| Pseudomonas aeruginosa | 1 |
| Raoultella planticola | 1 |
| Salmonella enterica | 1 |
| **total** | **262** |

**Table S3. Bacterial information for evolutionary analysis**

| **Index** | **Accession number** | **Species** |
| --- | --- | --- |
| 1 | gi\|1231143664\|gb\|CP022698.1\| | *Citrobacter farmeri* |
| 2 | gi\|1243044510\|gb\|MF497781.1\| | *Citrobacter freundii* |
| 3 | gi\|1493603797\|gb\|MH477637.1\| | *Citrobacter freundii* |
| 4 | gi\|1535846779\|gb\|MH919378.1\| | *Citrobacter freundii* |
| 5 | gi\|1243044469\|gb\|MF497780.1\| | *Citrobacter freundii* |
| 6 | gi\|1301643982\|gb\|MG557994.1\| | *Citrobacter freundii* |
| 7 | gi\|1301644023\|gb\|MG557995.1\| | *Citrobacter freundii* |
| 8 | gi\|1301644064\|gb\|MG557996.1\| | *Citrobacter freundii* |
| 9 | gi\|1301644105\|gb\|MG557997.1\| | *Citrobacter freundii* |
| 10 | gi\|1384232072\|dbj\|AP018567.1\| | *Citrobacter freundii* |
| 11 | gi\|1418454405\|gb\|CP030345.1\| | *Enterobacter cloacae* |
| 12 | gi\|787518906\|gb\|KM877517.1\| | *Enterobacter cloacae* |
| 13 | gi\|1004328143\|gb\|KU302802.1\| | *Enterobacter cloacae* |
| 14 | gi\|1004327978\|gb\|KU302801.1\| | *Enterobacter cloacae* |
| 15 | gi\|828895865\|gb\|CP011571.1\| | *Enterobacter cloacae* |
| 16 | gi\|828911534\|gb\|CP011580.1\| | *Enterobacter cloacae* |
| 17 | gi\|828977575\|gb\|CP011649.1\| | *Enterobacter cloacae* |
| 18 | gi\|1384662975\|gb\|MG764554.1\| | *Enterobacter cloacae* |
| 19 | gi\|828916727\|gb\|CP011583.1\| | *Enterobacter cloacae* |
| 20 | gi\|1395327219\|gb\|CP021168.1\| | *Enterobacter cloacae* |
| 21 | gi\|1395312997\|gb\|CP021163.1\| | *Enterobacter cloacae* |
| 22 | gi\|682112725\|gb\|CP008898.1\| | *Enterobacter cloacae* |
| 23 | gi\|1167690283\|gb\|KY296104.1\| | *Enterobacter cloacae* |
| 24 | gi\|1467491785\|dbj\|AP018830.1\| | *Enterobacter hormaechei* |
| 25 | gi\|1584097536\|gb\|CP036311.1\| | *Enterobacter hormaechei* |
| 26 | gi\|1376514255\|gb\|MG252895.1\| | *Escherichia coli* |
| 27 | gi\|1243045118\|gb\|MF589339.1\| | *Escherichia coli* |
| 28 | gi\|1360512703\|emb\|LT985249.1\| | *Escherichia coli* |
| 29 | gi\|1114159551\|gb\|CP010149.1\| | *Escherichia coli* |
| 30 | gi\|1190360403\|gb\|KY798506.1\| | *Escherichia coli* |
| 31 | gi\|938507360\|gb\|KT845955.1\| | *Escherichia coli* |
| 32 | gi\|1395893969\|gb\|CP021195.1\| | *Escherichia coli* |
| 33 | gi\|1114210311\|gb\|CP010174.1\| | *Escherichia coli* |
| 34 | gi\|1208619820\|gb\|CP021881.1\| | *Escherichia coli* |
| 35 | gi\|1384651626\|gb\|MG825381.1\| | *Escherichia coli* |
| 36 | gi\|1433039703\|gb\|CP025944.3\| | *Escherichia coli* |
| 37 | gi\|1151186796\|gb\|CP018989.1\| | *Escherichia coli* |
| 38 | gi\|1493765939\|gb\|CP032988.1\| | *Escherichia coli* |
| 39 | gi\|1301644187\|gb\|MG557999.1\| | *Escherichia coli* |
| 40 | gi\|1441520707\|gb\|MG836696.1\| | *Escherichia coli* |
| 41 | gi\|1279194805\|gb\|CP024857.1\| | *Escherichia coli* |
| 42 | gi\|1151231000\|gb\|CP019017.1\| | *Escherichia coli* |
| 43 | gi\|651033488\|emb\|HG963476.1\| | *Escherichia coli* |
| 44 | gi\|1108565077\|gb\|CP018105.1\| | *Escherichia coli* |
| 45 | gi\|1108569934\|gb\|CP018111.1\| | *Escherichia coli* |
| 46 | gi\|1108574783\|gb\|CP018117.1\| | *Escherichia coli* |
| 47 | gi\|1108579629\|gb\|CP018123.1\| | *Escherichia coli* |
| 48 | gi\|1360518725\|emb\|LT985322.1\| | *Escherichia coli* |
| 49 | gi\|218350150\|emb\|CU928144.1\| | *Escherichia fergusonii* |
| 50 | gi\|1376190040\|gb\|MG288683.1\| | *Klebsiella aerogenes* |
| 51 | pR50-74 | *Klebsiella pneumoniae* |
| 52 | gi\|1062713554\|gb\|CP012566.1\| | *Klebsiella pneumoniae* |
| 53 | gi\|1062719365\|gb\|CP012571.1\| | *Klebsiella pneumoniae* |
| 54 | gi\|1391531868\|gb\|MH255827.1\| | *Klebsiella pneumoniae* |
| 55 | gi\|365803828\|gb\|CP003224.1\| | *Klebsiella pneumoniae* |
| 56 | gi\|472302154\|gb\|KC405622.1\| | *Klebsiella pneumoniae* |
| 57 | gi\|1206209274\|gb\|CP021761.1\| | *Klebsiella pneumoniae* |
| 58 | gi\|1057968291\|gb\|CP016919.1\| | *Klebsiella pneumoniae* |
| 59 | gi\|1120824496\|gb\|CP018724.1\| | *Klebsiella pneumoniae* |
| 60 | gi\|1120800916\|gb\|CP018699.1\| | *Klebsiella pneumoniae* |
| 61 | gi\|1476650218\|gb\|CP032227.1\| | *Klebsiella pneumoniae* |
| 62 | gi\|1114568913\|gb\|CP018354.1\| | *Klebsiella pneumoniae* |
| 63 | gi\|1202218802\|gb\|CP021547.1\| | *Klebsiella pneumoniae* |
| 64 | gi\|1208699032\|gb\|CP021943.1\| | *Klebsiella pneumoniae* |
| 65 | gi\|1432252197\|dbj\|AP018755.1\| | *Klebsiella pneumoniae* |
| 66 | gi\|1519475198\|gb\|CP033901.1\| | *Klebsiella pneumoniae* |
| 67 | gi\|1384232088\|dbj\|AP018583.1\| | *Klebsiella pneumoniae* |
| 68 | gi\|1187402631\|gb\|CP020902.1\| | *Klebsiella pneumoniae* |
| 69 | gi\|1409373696\|gb\|CP022612.1\| | *Klebsiella pneumoniae* |
| 70 | gi\|530347473\|gb\|CP006657.1\| | *Klebsiella pneumoniae* |
| 71 | gi\|1114601939\|gb\|CP018365.1\| | *Klebsiella pneumoniae* |
| 72 | gi\|1574042019\|gb\|MK396843.1\| | *Klebsiella pneumoniae* |
| 73 | gi\|1339013836\|gb\|CP026397.1\| | *Klebsiella pneumoniae* |
| 74 | gi\|902611358\|gb\|CP011977.1\| | *Klebsiella pneumoniae* |
| 75 | gi\|1530713295\|gb\|CP034040.1\| | *Klebsiella pneumoniae* |
| 76 | gi\|1269242179\|gb\|CP023554.1\| | *Klebsiella pneumoniae* |
| 77 | gi\|1266999745\|gb\|CP023942.1\| | *Klebsiella pneumoniae* |
| 78 | gi\|1398622440\|gb\|CP029723.1\| | *Klebsiella pneumoniae* |
| 79 | gi\|1240181407\|gb\|MF437312.1\| | *Klebsiella pneumoniae* |
| 80 | gi\|1399734359\|gb\|CP028541.2\| | *Klebsiella pneumoniae* |
| 81 | gi\|943457735\|gb\|KT818627.1\| | *Klebsiella pneumoniae* |
| 82 | gi\|1332754599\|gb\|CP025952.1\| | *Klebsiella pneumoniae* |
| 83 | gi\|1233707932\|gb\|MF168403.1\| | *Klebsiella pneumoniae* |
| 84 | gi\|1233708340\|gb\|MF168405.1\| | *Klebsiella pneumoniae* |
| 85 | gi\|1384664493\|gb\|MF156695.1\| | *Klebsiella pneumoniae* |
| 86 | gi\|1444558613\|gb\|CP026131.1\| | *Klebsiella pneumoniae* |
| 87 | gi\|1564248914\|gb\|CP026141.1\| | *Klebsiella pneumoniae* |
| 88 | gi\|1384661323\|gb\|MG764550.1\| | *Klebsiella pneumoniae* |
| 89 | gi\|1220493807\|gb\|MF133495.1\| | *Klebsiella pneumoniae* |
| 90 | gi\|1486391278\|gb\|MH477636.1\| | *Klebsiella pneumoniae* |
| 91 | gi\|1114629524\|gb\|CP018455.1\| | *Klebsiella pneumoniae* |
| 92 | gi\|902625122\|gb\|CP011990.1\| | *Klebsiella pneumoniae* |
| 93 | gi\|1317758576\|gb\|CP025463.1\| | *Klebsiella pneumoniae* |
| 94 | gi\|1534487993\|gb\|CP034324.1\| | *Klebsiella pneumoniae* |
| 95 | gi\|1344162864\|gb\|CP026584.1\| | *Klebsiella pneumoniae* |
| 96 | gi\|1552773062\|gb\|MK167987.1\| | *Klebsiella pneumoniae* |
| 97 | gi\|1509152242\|gb\|CP033404.1\| | *Klebsiella pneumoniae* |
| 98 | gi\|1464473000\|gb\|CP031720.1\| | *Klebsiella pneumoniae* |
| 99 | gi\|1584047593\|gb\|CP036301.1\| | *Klebsiella pneumoniae* |
| 100 | gi\|1552773384\|gb\|MK167989.1\| | *Klebsiella pneumoniae* |
| 101 | gi\|1233708139\|gb\|MF168404.1\| | *Klebsiella pneumoniae* |
| 102 | gi\|961666360\|gb\|KT185451.1\| | *Klebsiella pneumoniae* |
| 103 | gi\|299474760\|gb\|FJ628167.2\| | *Klebsiella pneumoniae* |
| 104 | gi\|1378055449\|gb\|CP028582.1\| | *Klebsiella pneumoniae* |
| 105 | gi\|1390424610\|gb\|CP029381.1\| | *Klebsiella pneumoniae* |
| 106 | gi\|1233707742\|gb\|MF168402.1\| | *Klebsiella pneumoniae* |
| 107 | gi\|1388214979\|gb\|CP029225.1\| | *Klebsiella pneumoniae* |
| 108 | gi\|1212890037\|gb\|KY174332.1\| | *Klebsiella pneumoniae* |
| 109 | gi\|1382825734\|gb\|CP028955.1\| | *Klebsiella pneumoniae* |
| 110 | gi\|746616805\|gb\|KJ187752.1\| | *Klebsiella pneumoniae* |
| 111 | gi\|1511001896\|gb\|MK104259.1\| | *Klebsiella pneumoniae* |
| 112 | gi\|1360859627\|gb\|CP024430.1\| | *Klebsiella pneumoniae* |
| 113 | gi\|1378921531\|gb\|CP028805.1\| | *Klebsiella pneumoniae* |
| 114 | gi\|1317764187\|gb\|CP025468.1\| | *Klebsiella pneumoniae* |
| 115 | gi\|1317752866\|gb\|CP025458.1\| | *Klebsiella pneumoniae* |
| 116 | gi\|751372783\|gb\|KP008371.1\| | *Klebsiella pneumoniae* |
| 117 | gi\|1476622549\|gb\|CP032189.1\| | *Klebsiella pneumoniae* |
| 118 | gi\|1391531392\|gb\|MH263653.1\| | *Klebsiella pneumoniae* |
| 119 | gi\|1584087299\|gb\|CP036362.1\| | *Klebsiella pneumoniae* |
| 120 | gi\|1353787790\|gb\|CP027067.1\| | *Klebsiella pneumoniae* |
| 121 | gi\|1378926910\|gb\|CP028796.1\| | *Klebsiella pneumoniae* |
| 122 | gi\|1190361309\|gb\|KY751926.1\| | *Klebsiella pneumoniae* |
| 123 | gi\|1468960166\|gb\|CP031883.1\| | *Klebsiella pneumoniae* |
| 124 | gi\|1559984379\|gb\|MH917279.1\| | *Klebsiella pneumoniae* |
| 125 | gi\|1517371350\|gb\|CP033628.1\| | *Klebsiella pneumoniae* |
| 126 | gi\|1526233929\|gb\|CP034125.1\| | *Klebsiella pneumoniae* |
| 127 | gi\|406352863\|gb\|JX283456.1\| | *Klebsiella pneumoniae* |
| 128 | gi\|1035427824\|emb\|LT009689.1\| | *Klebsiella pneumoniae* |
| 129 | gi\|1095429891\|gb\|CP016403.1\| | *Klebsiella pneumoniae* |
| 130 | gi\|1564237462\|gb\|CP026133.1\| | *Klebsiella pneumoniae* |
| 131 | gi\|1352749782\|gb\|CP025213.1\| | *Klebsiella pneumoniae* |
| 132 | gi\|1398288122\|gb\|CP023725.1\| | *Klebsiella pneumoniae* |
| 133 | gi\|110264421\|gb\|DQ449578.1\| | *Klebsiella pneumoniae* |
| 134 | gi\|1028116776\|emb\|HG969996.1\| | *Klebsiella pneumoniae* |
| 135 | gi\|672605144\|gb\|CP009116.1\| | *Klebsiella pneumoniae* |
| 136 | gi\|1114620738\|gb\|CP018448.1\| | *Klebsiella pneumoniae* |
| 137 | gi\|1114626366\|gb\|CP018451.1\| | *Klebsiella pneumoniae* |
| 138 | gi\|1476639402\|gb\|CP032176.1\| | *Klebsiella pneumoniae* |
| 139 | gi\|1274894564\|gb\|CP024573.1\| | *Klebsiella pneumoniae* |
| 140 | gi\|1093006983\|gb\|CP011839.1\| | *Klebsiella pneumoniae* |
| 141 | gi\|640856169\|gb\|CP007735.1\| | *Klebsiella pneumoniae* |
| 142 | gi\|1100613181\|gb\|CP017935.1\| | *Klebsiella pneumoniae* |
| 143 | gi\|1206193013\|gb\|CP021741.1\| | *Klebsiella pneumoniae* |
| 144 | gi\|150958513\|gb\|CP000650.1\| | *Klebsiella pneumoniae* |
| 145 | gi\|1202259309\|gb\|CP021698.1\| | *Klebsiella pneumoniae* |
| 146 | gi\|1160548085\|gb\|KY689238.1\| | *Klebsiella pneumoniae* |
| 147 | gi\|660582175\|gb\|CP008798.1\| | *Klebsiella pneumoniae* |
| 148 | gi\|743008998\|gb\|CP010362.1\| | *Klebsiella pneumoniae* |
| 149 | gi\|971339514\|gb\|CP006662.2\| | *Klebsiella pneumoniae* |
| 150 | gi\|1125932667\|gb\|CP018819.1\| | *Klebsiella pneumoniae* |
| 151 | gi\|1564260351\|gb\|CP026152.1\| | *Klebsiella pneumoniae* |
| 152 | gi\|1552089775\|gb\|CP025578.1\| | *Klebsiella pneumoniae* |
| 153 | gi\|1266987875\|gb\|CP023926.1\| | *Klebsiella pneumoniae* |
| 154 | gi\|1266993934\|gb\|CP023934.1\| | *Klebsiella pneumoniae* |
| 155 | gi\|1317769507\|gb\|CP025517.1\| | *Klebsiella pneumoniae* |
| 156 | gi\|1383742965\|gb\|MG764549.1\| | *Klebsiella pneumoniae* |
| 157 | gi\|1036427838\|gb\|CP015991.1\| | *Klebsiella pneumoniae* |
| 158 | gi\|1196625196\|gb\|CP020856.1\| | *Klebsiella pneumoniae* |
| 159 | gi\|760459974\|gb\|KJ958927.1\| | *Klebsiella pneumoniae* |
| 160 | gi\|1163072007\|gb\|CP020110.1\| | *Klebsiella pneumoniae* |
| 161 | gi\|1123428128\|gb\|CP018737.1\| | *Klebsiella pneumoniae* |
| 162 | gi\|754341646\|gb\|CP010575.1\| | *Klebsiella pneumoniae* |
| 163 | gi\|742999823\|gb\|CP010396.1\| | *Klebsiella pneumoniae* |
| 164 | gi\|1450480291\|gb\|CP031581.1\| | *Klebsiella pneumoniae* |
| 165 | gi\|1380945488\|gb\|CP028931.1\| | *Klebsiella pneumoniae* |
| 166 | gi\|667713574\|gb\|CP006801.1\| | *Klebsiella pneumoniae* |
| 167 | gi\|1345404136\|gb\|CP026753.1\| | *Klebsiella pneumoniae* |
| 168 | gi\|1384494271\|gb\|CP029135.1\| | *Klebsiella pneumoniae* |
| 169 | gi\|1202265413\|gb\|CP021712.1\| | *Klebsiella pneumoniae* |
| 170 | gi\|1208693517\|gb\|CP021953.1\| | *Klebsiella pneumoniae* |
| 171 | gi\|1114563368\|gb\|CP018350.1\| | *Klebsiella pneumoniae* |
| 172 | gi\|1051446430\|gb\|CP016812.1\| | *Klebsiella pneumoniae* |
| 173 | gi\|1353813931\|gb\|CP027150.1\| | *Klebsiella pneumoniae* |
| 174 | gi\|1417734589\|emb\|LT994836.1\| | *Klebsiella pneumoniae* |
| 175 | gi\|571430855\|gb\|KF793937.1\| | *Klebsiella pneumoniae* |
| 176 | gi\|1336001450\|gb\|CP026182.1\| | *Klebsiella pneumoniae* |
| 177 | gi\|1110961487\|gb\|CP018316.1\| | *Klebsiella pneumoniae* |
| 178 | gi\|1130049140\|gb\|CP017989.1\| | *Klebsiella pneumoniae* |
| 179 | gi\|1120806789\|gb\|CP018705.1\| | *Klebsiella pneumoniae* |
| 180 | gi\|1120818617\|gb\|CP018718.1\| | *Klebsiella pneumoniae* |
| 181 | gi\|1120812666\|gb\|CP018711.1\| | *Klebsiella pneumoniae* |
| 182 | gi\|1120789574\|gb\|CP018691.1\| | *Klebsiella pneumoniae* |
| 183 | gi\|1464578621\|gb\|CP030134.1\| | *Klebsiella pneumoniae* |
| 184 | gi\|1243044413\|gb\|MF510424.1\| | *Klebsiella pneumoniae* |
| 185 | gi\|1417734845\|emb\|LT994839.1\| | *Klebsiella pneumoniae* |
| 186 | gi\|1518649432\|emb\|LT968753.1\| | *Klebsiella pneumoniae* |
| 187 | gi\|1509398265\|gb\|CP033395.1\| | *Klebsiella pneumoniae* |
| 188 | gi\|1417734670\|emb\|LT994837.1\| | *Klebsiella pneumoniae* |
| 189 | gi\|1034308689\|gb\|KU665641.1\| | *Klebsiella pneumoniae* |
| 190 | gi\|1530771818\|gb\|CP027047.1\| | *Klebsiella pneumoniae* |
| 191 | gi\|929916961\|gb\|KT225462.1\| | *Klebsiella pneumoniae* |
| 192 | gi\|512492521\|gb\|JX424614.1\| | *Klebsiella pneumoniae* |
| 193 | gi\|1384139278\|gb\|CP028993.1\| | *Klebsiella pneumoniae* |
| 194 | gi\|1417734121\|emb\|LT994834.1\| | *Klebsiella pneumoniae* |
| 195 | gi\|1567626728\|gb\|CP035538.1\| | *Klebsiella pneumoniae* |
| 196 | gi\|1207892797\|gb\|CP021858.1\| | *Klebsiella pneumoniae* |
| 197 | gi\|1301644146\|gb\|MG557998.1\| | *Klebsiella pneumoniae* |
| 198 | gi\|574609170\|gb\|KF954150.1\| | *Klebsiella pneumoniae* |
| 199 | gi\|1206198916\|gb\|CP021755.1\| | *Klebsiella pneumoniae* |
| 200 | gi\|1511002242\|gb\|MK079573.1\| | *Klebsiella pneumoniae* |
| 201 | gi\|1353857435\|gb\|CP027162.1\| | *Klebsiella pneumoniae* |
| 202 | gi\|828971497\|gb\|CP011644.1\| | *Klebsiella pneumoniae* |
| 203 | gi\|1163063542\|gb\|CP020073.1\| | *Klebsiella pneumoniae* |
| 204 | gi\|1150881362\|gb\|CP014299.1\| | *Klebsiella pneumoniae* |
| 205 | gi\|1040719028\|dbj\|LC155908.1\| | *Klebsiella pneumoniae* |
| 206 | gi\|1030066427\|gb\|CP014758.1\| | *Klebsiella pneumoniae* |
| 207 | gi\|1208687900\|gb\|CP021948.1\| | *Klebsiella pneumoniae* |
| 208 | gi\|1345511443\|gb\|CP014124.1\| | *Klebsiella pneumoniae* |
| 209 | gi\|1163055369\|gb\|CP020070.1\| | *Klebsiella pneumoniae* |
| 210 | gi\|1432246590\|dbj\|AP018752.1\| | *Klebsiella pneumoniae* |
| 211 | gi\|660560816\|gb\|CP008701.1\| | *Klebsiella variicola* |
| 212 | gi\|1378049846\|gb\|CP028551.1\| | *Klebsiella variicola* |
| 213 | gi\|1026616321\|gb\|CP015347.1\| | *Proteus mirabilis* |
| 214 | gi\|1391530997\|gb\|MH061383.1\| | *Pseudomonas aeruginosa* |
| 215 | gi\|1336438856\|gb\|CP019900.1\| | *Raoultella planticola* |
